# Rapid Acquisition of Linezolid Resistance in Methicillin-Resistant *Staphylococcus aureus*: Role of Hypermutation and Homologous Recombination

**DOI:** 10.1371/journal.pone.0155512

**Published:** 2016-05-16

**Authors:** Shigekazu Iguchi, Tomonori Mizutani, Keiichi Hiramatsu, Ken Kikuchi

**Affiliations:** 1 Department of Infection Control Science, Faculty of Medicine, Juntendo University, Tokyo, Japan; 2 Department of Microbiology, Faculty of Medicine, Juntendo University, Tokyo, Japan; 3 Department of Infectious Diseases, Tokyo Women`s Medical University, Tokyo, Japan; Rockefeller University, UNITED STATES

## Abstract

**Background:**

We previously reported the case of a 64-year-old man with mediastinitis caused by *Staphylococcus aureus* in which the infecting bacterium acquired linezolid resistance after only 14 days treatment with linezolid. We therefore investigated relevant clinical isolates for possible mechanisms of this rapid acquisition of linezolid resistance.

**Methods:**

Using clinical *S*. *aureus* isolates, we assessed the in vitro mutation rate and performed stepwise selection for linezolid resistance. To investigate homologous recombination, sequences were determined for each of the 23S ribosomal RNA (23S rRNA) loci; analyzed sequences spanned the entirety of each 23S rRNA gene, including domain V, as well as the 16S-23S intergenic spacer regions. We additionally performed next-generation sequencing on clinical strains to identify single-nucleotide polymorphisms compared to the N315 genome.

**Results:**

Strains isolated from the patient prior to linezolid exposure (M5-M7) showed higher-level linezolid resistance than N315, and the pre-exposure strain (M2) exhibited more rapid acquisition of linezolid resistance than did N315. However, the mutation rates of these and contemporaneous clinical isolates were similar to those of N315, and the isolates did not exhibit any mutations in hypermutation-related genes. Sequences of the 23S rRNA genes and 16S-23S intergenic spacer regions were identical among the pre- and post-exposure clinical strains. Notably, all of the pre-exposure isolates harbored a *recQ* missense mutation (Glu69Asp) with respect to N315; such a lesion may have affected short sequence recombination (facilitating, for example, recombination among *rrn* loci). We hypothesize that this mechanism contributed to rapid acquisition of linezolid resistance.

**Conclusions:**

Hypermutation and homologous recombination of the ribosomal RNA genes, including 23S rRNA genes, appear not to have been sources of the accelerated acquisition of linezolid resistance observed in our clinical case. Increased frequency of short sequence recombination may have resulted from a *recQ* variant in the infecting organism.

## Introduction

Linezolid (LZD) is the first approved antibiotic of the oxazolidinone class. This agent is effective for the treatment of infectious diseases caused by Gram-positive bacteria, including methicillin-resistant *Staphylococcus aureus* (MRSA). LZD inhibits protein synthesis by binding to domain V of the 23S rRNA, thereby preventing the formation of the functional 70S initiation complex required for the bacterial translation process [1]. Reported mechanisms of LZD resistance include point mutation of domain V of the 23S rRNA gene [2], mutation of the ribosomal proteins close to the linezolid binding site in the ribosomal peptidyl transferase center [3], and acquisition of *cfr*, which encodes a 23S rRNA methylase [4]. However, the clinical occurrence of LZD resistance is rare, and has been observed only following prolonged exposure to the drug [5]. This observation is consistent with the low frequency of in vitro mutation, reported as less than 8 × 10^−11^ [6]. Recently, we reported consecutive LZD-resistant *S*. *aureus* (LRSA) strains isolated from a patient’s blood cultures after only 2 weeks of LZD treatment [7]. The minimum inhibitory concentration (MIC) of LZD following exposure was 32 μg/ml, compared to an MIC of 4 μg/ml in the parent strain. These LRSA strains harbored a G2576T mutation in multiple 23S rRNA loci. While we could not explain the rapid acquisition of LZD resistance by these strains, the effect is reminiscent of a hypermutator phenotype. Hypermutator bacteria exhibit high frequencies of point mutations, often through the inactivation of mismatch repair genes, resulting in the rapid acquisition of antimicrobial resistance [8]. Separate work has suggested a role for homologous recombination in the spread of domain V mutations among the multiple copies of the 23S rRNA genes in a given genome [9, 10], although this proposed mechanism was not demonstrated directly. In the present study, we attempted to elucidate the mechanism(s) of the rapid acquisition of LZD resistance in our clinical isolates of *S*. *aureus*.

## Materials and Methods

### Bacterial strains and culture condition

All clinical strains are listed in Table 1. Four MRSA or LRSA strains (designated M2, M5, M6, and M7) were consecutively isolated from the same patient before and after use of LZD [7]. M6 is a small colony variant (SCV) that exhibited hemin auxotrophy [7]. Other clinical isolates (M3, M4, M30, M32, and M13) were obtained as described in Table 1. Each strain was subcultured on TSA with 5% sheep blood (Kohjin Bio Co. Ltd., Saitama, Japan) at 37°C for 24 hours. Mu3, Mu50, and N315 complete genome data were obtained from GenBank as accession numbers AP009324, BA000017.4, and BA000018, respectively.

**Table 1:** List of strains used.

Strain	References	Comments	State	MIC^a^ (μg/ml) of							MLST^b^	PFGE^c^
No.			of infection	VAN	GEN	ABK	TET	CHL	RIF	LZD		Type
**M2**	**7**	**Case 1, before use of LZD**	Infection	1	>32	4	1	8	0.03	4	ST5	A1
**M5**	**7**	**Case1, after use of LZD**	Infection	1	>32	8	1	32	0.03	32	ST5	A1
**M6**	**7**	**Case1, after use of LZD, SCV**	Infection	1	>32	8	1	32	0.03	32	ST5	A1
**M7**	**7**	**Case1, after use of LZD**	Infection	1	>32	2	1	32	0.03	32	ND^d^	A1
**M3**	**7**	**Case 3**	Infection	1	>32	4	1	8	0.03	4	ST5	A1
**M4**	**7**	**Case 4**	Infection	1	>32	1	1	8	0.03	4	ST5	A1
**M30**	**7**	**Case 7**	Carriage	0.5	>32	1	1	8	0.03	4	ND^d^	A1
**M32**	**7**	**Case 8**	Carriage	1	>32	2	1	8	0.03	4	ND^d^	A1
**M13**	**7**	**Case 14**	Carriage	2	<1	1	>16	8	0.03	2	ND^d^	C1
**N315**	**7**	**HA-MRSA prototype**		ND^d^	ND^d^	ND^d^	ND^d^	8	0.03	2	ST5	ND^d^

^a^VAN, Vancomycin; GEN, Gentamicin; ABK, Arbekacin; TET, Tetracycline; CHL, Chloramphenicol; RIF, Rifampicin; LZD, Linezolid

^b^MLST: Multi Locus Sequence Typing

^c^PFGE: pulsed-field gel electrophoresis

^d^ND: Not done

### Antimicrobial susceptibility test

The MICs were determined by the agar plate dilution method; the interpretation of these results was performed in accordance with Clinical Laboratory Standards [11].

### Stepwise LZD resistance selection assay

Individual colonies of M2, M3, M13, and N315 were resuspended in sterile saline at 0.5 McFarland standard and 10 μl of each suspension was inoculated in 1 ml brain heart infusion (BHI) broth (Nippon Beckton-Dickinson, Fukushima, Japan) containing LZD at concentrations of 0, 0.5, 1, 2, 4, 8, 16, 32, or 64 μg/ml. All cultures were incubated at 37°C for 24 hours with vigorous shaking (200 rpm/min). For each strain, an aliquot (10 μl) of the culture with the maximum LZD concentration that still showed visible turbidity was inoculated into fresh BHI broth containing LZD as described above. This consecutive stepwise LZD resistance selection test was continued for a total of up to 32 passages. The resulting strains are designated using the format parent strain/resulting MIC/passage number.

At each MIC determination step, 1 loopful (approximately 1 μl) of culture from the well at 1/2 MIC was streaked to TSA with 5% sheep blood and incubated at 37°C for 24 hours. The resulting isolated colonies were used for PCR to generate templates for sequencing of the 23S rRNA (domain V) and 16S-23S IGS (intergenic spacer) regions.

### Doubling time

Test strains were incubated in BHI broth at 37°C for 20 hours; the resulting cultures were diluted to an optical density (OD) (at 578 nm) of 0.3 using BHI broth. An aliquot (100 μl) of each dilution was used to inoculate 10 ml BHI broth in a test tube, and the resulting cultures were grown using a TVS120MB (ADVANTEC, Tokyo, Japan) at 37°C with shaking at 25 rpm; the OD (at 600 nm) of each culture was measured automatically every 2 minutes for up to 249 hours. The resulting values were fitted to log approximate equations calculated as y = a * log x + b, where x = OD and y = time. The doubling times in the logarithmic growth phase were calculated as follows: doubling time = (b + a * log OD at t2)–(b + a * log OD at t1) = a * (log OD at t2—log OD at t1), where OD at t2 = 2 * OD at t1.

### Mutation rate

Individual colonies of test strains were resuspended in sterile saline at 0.5 McFarland. Aliquots (10 μl/inoculum) of these suspensions were used to inoculate 10 tubes containing 1 ml BHI broth each (small culture system) or 1 tube containing 2 ml BHI broth (big culture system). The resulting cultures were incubated at 37°C for 24 hours with shaking. For the small culture system, 100 μl aliquots of each of the 10 cultures were plated onto separate BHI agar (Nippon Beckton-Dickinson) plates containing 128x MIC of rifampin; for the large culture system, 100 μl aliquots of the culture were plated onto 10 separate BHI agar plates containing 128x MIC of rifampin. To determine the total number of colony forming units (CFUs), each culture was subjected to 10 fold serial dilutions, and 10 μl aliquots of each dilution were plated to BHI agar plates. After a 24-hour incubation at 37°C, the colonies were counted and mutation rate was calculated based on the Ma-Sandri-Sarkar Maximum Likelihood Estimator (MSS-MLE) method (Fluctuation AnaLysis CalculatOR, http://www.keshavsingh.org/protocols/FALCOR.html) [12]. Hypermutators were defined as strains that exhibited mutation rates at least 10 fold higher than those of control strains.

### DNA extraction

Individual colonies were resuspended in 100 μl Tris-EDTA (TE) buffer with 40 U of achromopeptidase (Wako Chemical, Kyoto, Japan). The suspensions were incubated 55°C for 15 minutes and then centrifuged at 20,000 x g at 4°C for 5 minutes, and the resulting supernatants were used for PCR amplification and sequencing of genomic DNA. For next-generation sequencing using the HiSeq2000 (Illumina, Inc., USA), cell lysates were processed using QIAprep Spin Miniprep Kits (QIAGEN, Germany) according to the manufacturer’s recommendations.

### 23S rRNA and 16S-26S intergenic spacer region sequencing

Each copy of 23S rRNA (*rrn*1—*rrn*5) gene was amplified using long PCR. The primer pairs were as previously described by Meka et al. [5]. PrimeSTAR GXL DNA Polymerase (Takara Bio Inc., Japan) was used for long PCR, and PCR conditions were as follows; denaturation at 95°C for 1 min; 32 PCR cycles consisting of denaturation at 94°C for 30 sec, annealing at 55°C for 30 sec, and extension at 68°C for 7 min; and final extension at 68°C for 10 min. PCR products were confirmed by 1% agarose gel electrophoresis. Table 2 shows the sequences of the primers used for sequencing of the full-length 23S rRNA genes; these primers were designed using the published genome for *S*. *aureus* N315 (GenBank accession no. NC_002745). For sequencing PCR of the domain V region of the 23S rRNA genes, we used 2 primers as previously published (GenBank accession no. X68425) [13]. For 16S-23S intergenic spacer (IGS) region sequencing, primers were as shown in Table 2. Sequencing reactions using BigDye Terminator v3.1 (Applied Biosystems, Life Technologies, Inc.) were performed as follows: preincubation at 95°C for 1 min; 32 cycles of denaturation at 94°C for 30 sec, annealing at 55°C for 30 sec, and extension at 72°C for 1 min; and final extension at 72°C for 3 min. The resulting products were analyzed on a 3730 DNA Analyzer (Applied Biosystems, Life Technologies, Inc.). Determination of 16S-23S IGS region typing was performed as previously reported [14].

**Table 2:** Primers used in this study.

Name	Target	Use	Sequence
***rrn*- Fw1**	**23S rRNA gene**	**Sequencing**	**5’-TTCGAAAGAACACTCACAAG-3’**
***rrn*- Fw2**	**23S rRNA gene**	**Sequencing**	**5’-GGACGACATTAGACGAATCATCTGG-3’**
***rrn*- Fw3**	**23S rRNA gene**	**Sequencing**	**5’-GTGAGCGGATGAACTGAGG-3’**
***rrn*- Fw4**	**23S rRNA gene**	**Sequencing**	**5’-GCGTTGAAGCATGATCGTAAG-3’**
***rrn*- Fw5**	**23S rRNA gene**	**Sequencing**	**5’-CTCGTTAAGGAACTCGGCA-3’**
***rrn*- Fw6**	**23S rRNA gene**	**Sequencing**	**5’-TCGGCACAGCTTGTACAGG-3’**
***rrn*- Fw7**	**23S rRNA gene**	**Sequencing**	**5’-ATCCTGGGGCTGTAGTCGG-3’**
***rrn*-Rv1**	**23S rRNA gene**	**Sequencing**	**5’-GACAACATTTTCGACTACAGG-3’**
***rrn*-Rv2**	**23S rRNA gene**	**Sequencing**	**5’-GAGAACCAGCTATCTCCAGG-3’**
***rrn*-Rv3**	**23S rRNA gene**	**Sequencing**	**5’-GTCTTTCGCTACTCACACCGGC-3’**
***rrn*-Rv4**	**23S rRNA gene**	**Sequencing**	**5’-GGCCTATTCACTGCGGCTC-3’**
***rrn*-Rv5**	**23S rRNA gene**	**Sequencing**	**5’-CCACGTAAGCTAGCGCTCACG-3’**
***rrn*-Rv6**	**23S rRNA gene**	**Sequencing**	**5’-CTGTCTCACGACGTTCTGAA-3’**
***rrn*-Rv7**	**23S rRNA gene**	**Sequencing**	**5’-CCTTGCTATAGTCACCAGAC-3’**
**IGS-F**	**16S-23S IGS region**	**Sequencing**	**5’-AGGAGCTAGCCGTCGAAGGT-3’**
**IGS-R**	**16S-23S IGS region**	**Sequencing**	**5’-AGCTGTAAGTAAGCTTTGATC-3’**

### M2 genome analysis and single-nucleotide polymorphism (SNP) phylogenetic tree

Shotgun sequencing of the M2 chromosomal DNA was performed using Illumina HiSeq 2000 (single reads) and Roche 454 (8-kb paired-end library). All reads were incorporated into a hybrid assembly using Newbler v2.6. Scaffolds were scanned for open reading frames (ORFs) and annotated using RAST [15] and BLAST [16].

The resulting M2 chromosome scaffold was compared to the published ST5 *S*. *aureus* reference complete genomes (N315, Mu3, Mu50) using snpTree-1.1 [17] to construct a SNP phylogenetic tree. We compared the M2 and N315 genomes for homologs using BlastP bidirectional best hit analysis with an identity threshold of 60% and an E-value threshold of 1E-08.

### SNP detection

We performed single-read sequencing analysis of six of our clinical strains (M2, M3, M5, M6, M7, M13) using HiSeq 2000 (Illumina, Inc., USA) and mapping based on the N315 genome (GenBank accession no. NC_002745) [18]; SNP lists were assembled using Genome Traveler (In Silico Biology, Inc., Mishima, Japan). We especially noted SNPs in hypermutation-related genes, focusing on previously reported loci [19, 20, 21, 22, 23, 24, 25, 26, 27, 28].

## Results

### Antimicrobial susceptibility test

The RIF MICs of all the tested strains (M2, M3, M4, M5, M6, M7, M13, M30, M32, N315) were 0.03 μg/ml.

### Stepwise LZD resistance selection test

**Fig 1:**
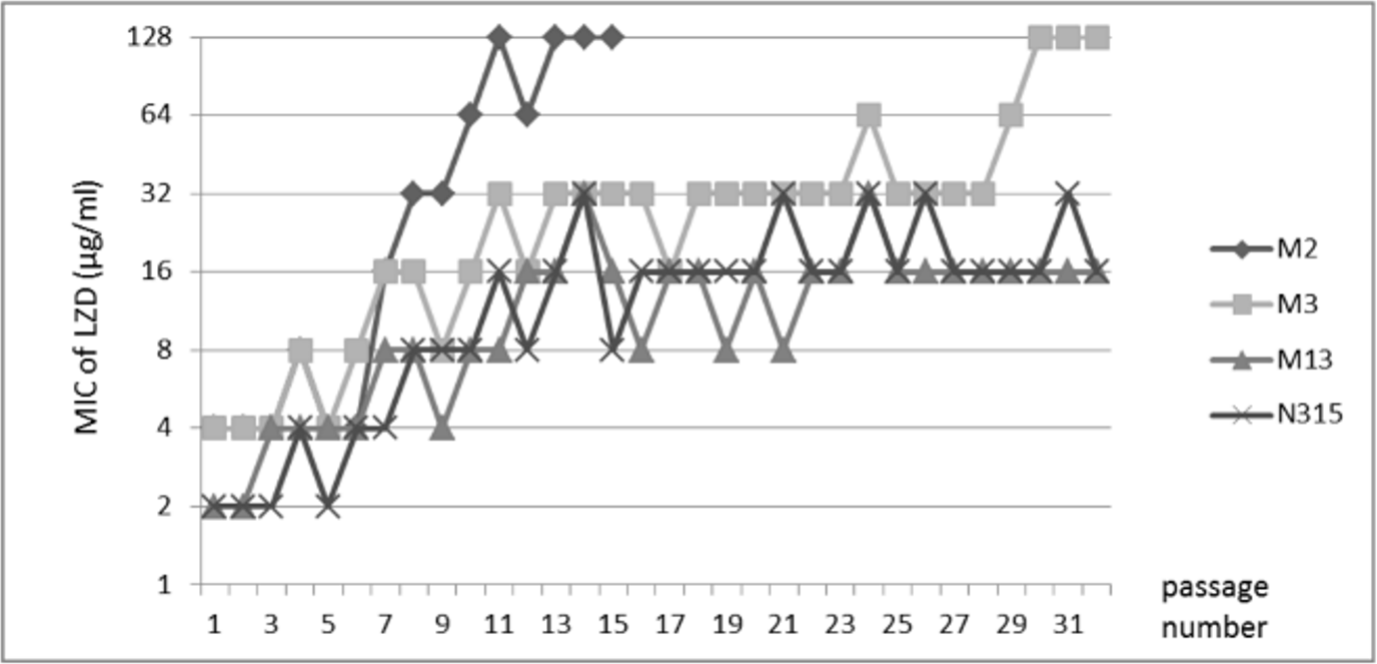
Stepwise selection of LZD-resistant mutants of MRSA strains. Strains M2 and M3 achieved MICs over 32 μg/ml in the short term, and reached 128 μg/ml by the end of the study. Strains M13 and N315 were used as controls in this experiment; neither strains achieved MICs of 32 μg/ml for two consecutive passages.

The results of stepwise LZD resistance selection tests are shown in Fig 1. Elevation of MIC was defined as hold of increased MIC at two or more consecutive passage. The MICs of LZD against M2 and M3 exceeded 32 μg/ml within 13 passages, while MICs continued to vary, values did not subsequently decrease more than 2-fold during passaging before finally reaching 128 μg/ml. In contrast, increases of the LZD MIC in strains M13 and N315 were relatively slow, peaking at 16 μg/ml after 12 passages and not rising higher even during long-term (over 30 times) passaging.

### Doubling time and mutation rate

Results of doubling time assays are shown in Table 3. N315 had the fastest doubling time; M6 had the slowest doubling time. Doubling times of the LRSA strains tended to be longer than those of the MRSA strains.

**Table 3:** Mutation rate and doubling time of strains used.

Strain	Mutation rate (X 10^−8^ CFU/ml) in small culture system		Mutation rate (X 10^−8^ CFU/ml) in big culture system		Doubling Time	
No.	Estimated	95% confidence interval	Estimated	95% confidence interval	Log Approximate Equation**	Doubling Time
		lower-upper bound		lower-upper bound		(minites)
**M2**	1.409	0.926–1.963	5.419	3.544–7.576	y = 38.938ln(x) + 247.48, R² = 0.9963	27
**M5**	2.008	1.391–2.705	7.884	5.129–11.056	y = 41.943ln(x) + 277.1, R² = 0.995	29.1
**M6**	1.45	0.964–2.006	14.453	10.455–18.912	y = 45.37ln(x) + 297.94, R² = 0.9977	31.4
**M7**	1.145	0.782–1.557	4.158	2.738–5.788	y = 43.382ln(x) + 290.73, R² = 0.9948	30.1
**M3**	0.802	0.49–1.167	4.542	3.13–6.139	y = 39.065ln(x) + 248.48, R² = 0.996	27.1
**M4**	1.559	1.037–2.157	2.36	1.434–3.446	y = 42.974ln(x) + 247.79, R² = 0.995	29.8
**M30**	1.307	0.899–1.769	1.552	0.937–2.276	y = 40.621ln(x) + 242.4, R² = 0.9974	28.2
**M32**	1.373	0.932–1.873	2.548	1.632–3.608	y = 37.004ln(x) + 252.56, R² = 0.9973	25.6
**M13**	0.596	0.372–0.857	1.526	1.025–2.099	ND*	ND*
**N315**	2.287	1.494–3.198	4.432	2.827–6.29	y = 32.128ln(x) + 221.05, R² = 0.9985	22.3

*ND; not done

** ln: natural logarithm, x: optical density, R²: coefficient determination

In the only big culture assay performed, M6 (SCV hemin auxotroph) had a significantly higher mutation rate than those of all the other strains, except for M5 (Table 3). However, none of the strains fulfilled the definition of hypermutator status.

### Intrachromosomal variations of 23S rRNA genes and characteristics of parent strains and isogenic mutants isolated from stepwise LZD resistance selection tests

*Staphylococcus aureus* have 5 or 6 copies of 23S rRNA genes with several intrachromosomal sequence variations (Table 4). Sequencing revealed that M2 and M3 harbored 3 SNPs in 1 copy of the 23S rRNA genes; the remaining 4 *rrn* loci in each genome had sequences identical to each other and to those of the reference genome. Specifically, *rrn2* carried C280A, C441T, and T1584A substitutions (all *S*. *aureus* numbering) compared to the reference genome. The *rrn* sequences of strain M2/128/15 (parent strain/MIC/passage number, i.e., a strain derived from M2 after 15 passages with LZD) were identical to those of the parent, except for the presence (in all 5 loci) of a G2576T (*E*. *coli* numbering) mutation.

**Table 4:** Intrachromosomal variations of 23S rRNA genes.

Strain	Intrachromosomal sequence variatios of 23S rRNA genes at respective positions										
	48	223	261	280	307	312	428	441	1584	2261	2576
**N315**	G	G	C	C, A (*rrn*2)^a^	A	A, G (*rrn*1)^a^	G	C	T, A(*rrn*1, 2)^a^	G, A (*rrn*1)^a^	G
**Mu50**	G	G	C	C, A (*rrn*2)^a^	A, G (*rrn*2)^a^	A, G (*rrn*3)^a^	G	C	T, A(*rrn*1, 2)^a^	G, A (*rrn*1)^a^	G
**M2, M3**	G	G	C	C, A (*rrn*2)^a^	A	A	G	C, T (*rrn*2)^a^	T, A (*rrn*2)^a^	G	G
**M2/128/15^b^**	G	G	C	C, A (*rrn*2)^a^	A	A	G	C, T (*rrn*2)^a^	T, A (*rrn*2)^a^	G	T (*rrn*1~5)

^a^: majority, minority base (minority copy number)

^b^: parent strain/LZD MIC/passage number (acquiring in step wise test)

Table 5 shows the relationship between the number of copies of the mutated 23S rRNA domain V gene and the MICs of LZD in strains derived in the stepwise selection. M2/128/15 had a G2576T (*E*. *coli* numbering) mutation in all *rrn* loci, and M3/128/32 had a G2447T (*E*. *coli* numbering) mutation in 3 *rrn* loci. Among the LZD-resistant mutants selected by stepwise selection from our clinical strains, the MIC of LZD tended to rise as the number of domain V mutation copies increased. However, LZD-resistant mutants derived by LZD selection from M13 or N315 harbored a G2236T (*S*. *aureus* numbering) mutation in only 1 of the *rrn* loci.

**Table 5:** Relationship of the presence of mutated domain V in the 23S rRNA genes with the level of LZD resistance

Parent strain	Domain V mutation in the 23S rRNA gene copies of each LZD-sensitive and -resistant strains at MIC of LZD						Mutational
(wild type)	4	8	16	32	64	128	site
**M2**	ND^a^ (6)^b^	*rrn*1, 4, 5 (7)^b^		*rrn*1,3,4,5 (10)^b^		*rrn*1,2,3,4,5 (15)^b^	G2576T
**M3**		ND^a^ (6)^b^	ND^a^ (8)^b^	ND^a^ (15)^b^	*rrn*1,4,5 (29)^b^	*rrn* 3,4,5 (32)^b^	G2447T
**M13**	*rrn*1 (6)^b^	*rrn1* (8)^b^	*rrn*1 (32)^b^				G2236T
**N315**	*rrn*1 (7)^b^	*rrn*1 (10)^b^	*rrn*1 (32)^b^				G2236T

^a^ ND: not detected domain V mutation

^b^ passage

### Changes of IGS region combinations in parent strains and isogenic LZD-resistant mutants

Table 6 shows 16S-23S IGS region sequences of the parent strains and the isogenic LZD-resistant mutants isolated during the stepwise LZD resistance selection. M2, M3, and Mu50 had the same sequences for the 16S-23S IGS regions. The IGS sequences at *rrn*1 to *rrn*5 were identical among M2, M3, and their isogenic mutants.

**Table 6:** Pattern of 16S-23S IGS regions in parental strains and isogenic linezolid (LZD)-resistant mutants.

Strain	Type of 16S-23S IGS				
	*rrn1*	*rrn2*	*rrn3*	*rrn4*	*rrn5*
**M2**	***rrnH***	***rrnJ***	***rrnC***	***rrnE***	***rrnE***
**M2/32/10^a^**	***rrnH***	***rrnJ***	***rrnC***	***rrnE***	***rrnE***
**M2/128/15^a^**	***rrnH***	***rrnJ***	***rrnC***	***rrnE***	***rrnE***
**M3**	***rrnH***	***rrnJ***	***rrnC***	***rrnE***	***rrnE***
**M3/64/29^a^**	***rrnH***	***rrnJ***	***rrnC***	***rrnE***	***rrnE***
**M3/128/32^a^**	***rrnH***	***rrnJ***	***rrnC***	***rrnE***	***rrnE***
**N315**	***rrnC***	***rrnJ***	***rrnC***	***rrnE***	***rrnE***
**Mu50**	***rrnH***	***rrnJ***	***rrnC***	***rrnE***	***rrnE***
**NCTC 8325**	***rrnH***	***rrnH***	***rrnC***	**A48073**	***rrnF***
**MRSA252**	***rrnY***	***rrnF***	***rrnC***	***rrnH***	***rrnZ***

^a^: wild type/LZD MIC/passage number (acquiring strains in step wise test) described by Tsuru T et al (ref 27).

### M2 genome analysis and SNP phylogenetic tree

**Fig 2:**
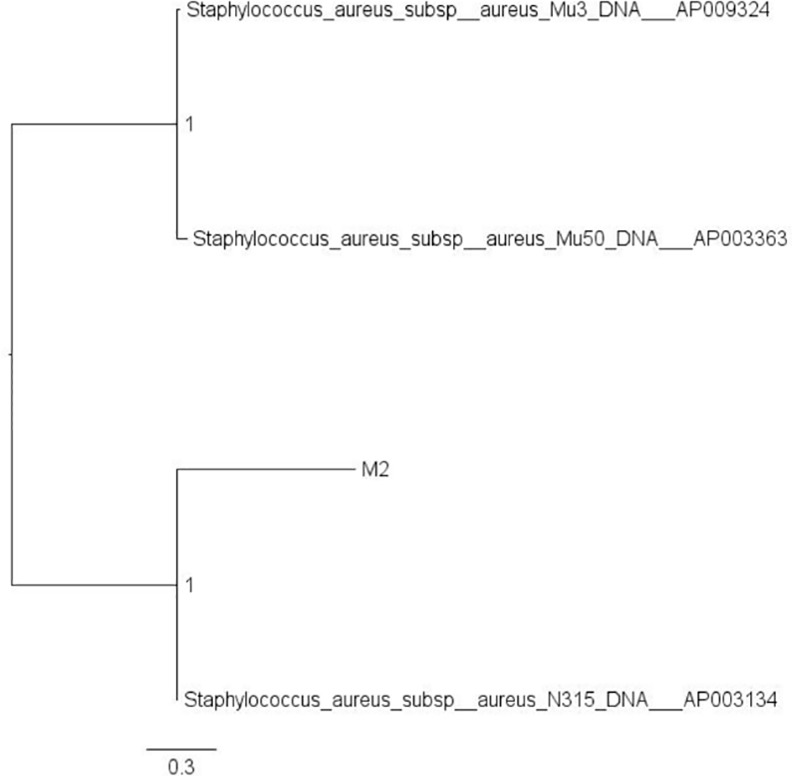
Phylogenetic tree of SNPs in the M2 and ST5 (MLST) reference strains.

Genomic sequence of M2 yielded 2 separate scaffolds. One scaffold corresponded to the circular chromosomal genome; the second scaffold corresponded to a circular plasmid. The scaffolds and annotation results are registered in DDBJ under accession numbers DF830066 (M2 chromosome genome) and DF830067 (M2 plasmid). A SNP phylogenetic tree derived by comparison to the ST5 strains is shown in Fig 2. N315 is the strain most closely related to M2.

**Fig 3:**
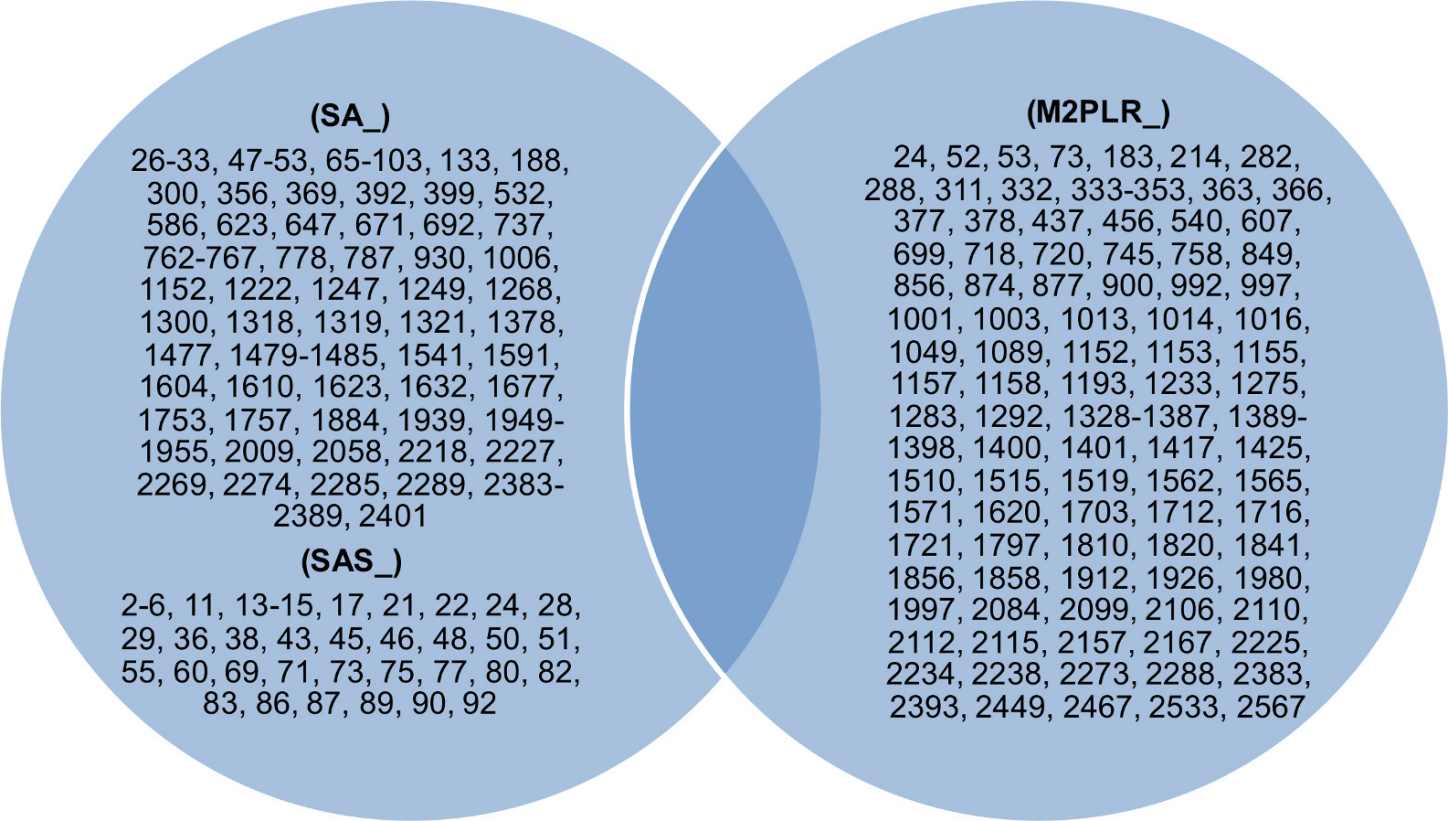
Venn diagram showing unique genes between N315 and M2 (SA or M2PLR number).

Genes unique to N315 and M2 are shown in Fig 3. Of the genes unique to M2 or N315, no genes are involved in DNA replication or repair system.

### SNP detection

We screened the sequences of over 20 genes previously reported [26, 27, 28] to contribute to hypermutation (for example, *mutS*, *mutL*, *uvrD*, and *dnaQ*). Analysis of the genomes of M2, M3, M5, M6, M7, and M13 revealed that none of these loci exhibited sequence variation compared to N315. However, in screening other loci known to be involved in DNA replication and repair, we found that only the *recQ* genes of M2, M3, M5, M6, and M7 (but not that of M13) harbored a missense mutation (Glu69Asp) compared to the reference genome, indicating that *recQ* is involved in DNA replication, repair, and recombination. None of the *rpl* gene mutations previously implicated in LZD resistance [3, 29] were detected (*rplC*, *rplD*, *rplV*). Strains M2, M3, M5, M6, and M7 (but not M13) harbored SNPs (compared to the reference genome) in *rplA* (which encodes ribosomal protein L1) and *rplR* (which encodes ribosomal protein L18). However, the L1 and L18 proteins are located far from the known linezolid-binding position [30, 31]; we therefore infer that these variants do not play a role in LZD resistance. Nonsense mutation of *hemH* (a T-to-A substitution at base 7 of the ORF, creating a premature termination codon) appeared responsible for the SCV and hemin auxotrophy phenotypes of M6 [32].

## Discussion

Known mechanisms of LZD resistance are mutation of a 23S rRNA gene (G2576T, T2500A, C2461T, or G2447T); *cfr* or *fexA* acquisition [33], and mutation of selected *rpl* loci (*rplC*, *rplD*, or *rplV*). In a systematic review, the most popular mechanism of linezolid resistance was G2576T mutation in 40 of 63 (63.5%) LRSA strains [34], as well as those in this study. Interestingly, these our clinical LRSA strains acquired the most frequently detected *rrn* mutation (G2576T) in three *rrn*s after only 2 weeks of clinical exposure to LZD, in contrast of acquisition of LZD resistance after 20 to 48.9 months administration of LZD [34, 35].

First, we focused on hypermutator-related genes such as *mut* directing mismatch repair system. Biser et al. reported that *S*. *aureus* SCV strains detected from sputum of cystic fibrosis patients exhibited resistance to GEN, fosfomycin, RIF, ciprofloxacin (CIP), and ERY [22]. Those *S*. *aureus* SCV strains harbored frame shift mutations in the *mutL* or *mutS* loci and exhibited elevated mutation frequencies. ERY resistance in those strains was due to A2058G and A2058T mutation in one or more 23S rRNA genes. This phenomenon suggests that hypermutator strains can more readily acquire point mutations in the 23S rRNA genes. Similarly, Oliver et al. reported that a *mutS*-deleted *Pseudomonas aeruginosa* exhibited an elevated mutation rate, and that the MIC and the minimum bactericidal concentration (MBC) of imipenem and CIP rose approximately 10-fold after a 36-hour exposure to these agents [21]. In contrast, in the present work, the mutation rates of our clinical isolates (both the original LZD-sensitive strains and the LZD-resistant strains) were similar to those determined for M13 and N315. However, we cannot define M6 as a hypermutator in our experiment, because the M6 strain exhibited only a 3-fold elevation in frequency of mutation to RIF resistance when compared to a reference strain. Additionally, this difference was not reproducible in several other experiments. Mapping of the M2, M3, M5, M6, and M7 genomes to the N315 genome revealed no other mutations at any previously described hypermutation-related genes.

Second, we considered the potential contribution of intrachromosomal recombination to LZD resistance. If a single domain V mutation occurs, the lesion could spread among multiple copies of 23S rRNA genes via intrachromosomal homologous recombination [9, 10]. Indeed, increases in the number of G2576T-mutated *rrn* loci correlated with increased LZD resistance in our strains. However, homologous recombination among mutated 23S rRNA gene copies has not been demonstrated directly in any articles. Therefore, we focused on 23S rRNA SNPs and 16S-23S IGS region sequences in our strains, compared with the 23S rRNA gene sequences published for 4 reference strains. These analyses revealed the presence of several intrachromosomal 23S rRNA variations and 16S-23S IGS regions in each ribosomal operon [18, 36, 37, 38]. The 16S-23S IGS regions include tRNA gene sequences and are composed of 13 variable sequences and 3 conserved sequences (CS). Over 10 distinct combination patterns of 16S-23S IGS regions have been reported, and have been explained as combinations of upper and lower variable sequences [14]. The possibility of homologous recombination involving the long homology of CS1 and CS2 in 16S-23S IGS region has been reported [38]. If homologous recombination among large fragments containing *rrn*s occurs, intrachromosomal exchanges of 23S rRNA sequences may occur, and 16S-23S IGS regions may be rearranged. Nevertheless, we did not detect any changes of intrachromosomal 23S rRNA variations or 16S-23S IGS regions between the parent strains and isogenic mutants characterized in the present study. However, we could not exclude the occurrence of very short sequence recombinations for regions encompassing domain V.

Third, as a result of SNP screening, we detected a missense mutation in the *recQ* gene (ORF M2PLR_1323 in our M2 genome sequence). The RecQ protein, which is conserved from bacteria to humans, is involved in the maintenance of genetic integrity; loss of function of the human RecQ homolog (BLM-helicase) is responsible for several human genetic disorders, including Bloom syndrome, Werner syndrome, and Rothmund-Thomson syndrome [39]. These diseases are characterized by hyper-recombination, genome rearrangements, and propensity to the development of cancer [39]. In *E*. *coli*, RecQ suppresses illegitimate recombination [40, 41]. Grierson et al. reported that deficiency for the BLM-helicase (Bloom syndrome RecQ like helicase) activity increased intrachromosomal recombination of rDNA repeats [42]. If the function of *recQ* in *S*. *aureus* is similar to that in *E*. *coli*, *recQ* mutation might increase intrachromosomal recombination among *rrn* loci, facilitating the rapid spread of domain V mutations among *rrn*s after acquisition of the first mutation. Further studies of the biological role of *recQ* in *S*. *aureus* will be needed.

23S rRNA gene mutation, *rpl* SNPs, and nonsense mutation of *hemH* may all be causes of slow growth. The doubling time tends to lengthen with increasing number of these factors. 23S rRNA is essential for maintaining the fundamental life process because of its role in protein synthesis. It has been reported that 23S A2503U, U2504G, G2505A, and G2576U mutations result in increased doubling times in *Mycobacterium smegmatis* [43, 44], with the G2576U mutation resulting in 3-fold slower growth [45]. Mutations in the *rplD* and *rplV* genes also have been shown to reduce growth rates [45, 46]. Although no mutations in *rplC*, *rplD*, or *rplV* were detected in our strains, *rplA rplR* mutations detected may affect slow growth rates. In the context of the present work, we hypothesize that these mutations may allow rapid selection of LZD-resistant mutants by permitting bacteria to slow protein synthesis and thereby escape LZD’s action.
